# Changes in the Second Ventilatory Threshold Following Individualised versus Standardised Exercise Prescription among Physically Inactive Adults: A Randomised Trial

**DOI:** 10.3390/ijerph19073962

**Published:** 2022-03-26

**Authors:** Alex D. Martini, Lance C. Dalleck, Gaizka Mejuto, Trent Larwood, Ryan M. Weatherwax, Joyce S. Ramos

**Affiliations:** 1 SHAPE Research Centre, Caring Futures Institute, Clinical Exercise Physiology, College of Nursing and Health Sciences, Flinders University, Adelaide, SA 5042, Australia; mart0790@flinders.edu.au (A.D.M.); ldalleck@western.edu (L.C.D.); trent.larwood@flinders.edu.au (T.L.); 2 Recreation, Exercise, and Sport Science Department, Western Colorado University, Gunnison, CO 81231, USA; 3 Faculty of Education, University of the Basque Country, 48940 Leioa, Spain; gaizka.mejuto@ehu.eus; 4 Health and Kinesiology Department, University of Utah, Salt Lake City, UT 84112, USA; rweatherwax@western.edu

**Keywords:** ventilatory threshold, individualised exercise prescription, standardised exercise prescription, cardiovascular disease, cardiovascular health, physically inactive

## Abstract

The second ventilatory threshold (VT_2_) is established as an important indicator of exercise intensity tolerance. A higher VT_2_ allows for greater duration of higher intensity exercise participation and subsequently greater reductions in cardiovascular disease (CVD) risk. This study aimed to compare the efficacy of standardised and individualised exercise prescription on VT_2_ among physically inactive adults. Forty-nine physically inactive male and female participants (48.6 ± 11.5 years) were recruited and randomised into a 12-week standardised (*n* = 25) or individualised (*n* = 24) exercise prescription intervention. The exercise intensity for the standardised and individualised groups was prescribed as a percentage of heart rate reserve (HRR) or relative to the first ventilatory threshold (VT_1_) and VT_2_, respectively. Participants were required to complete a maximal graded exercise test at pre-and post-intervention to determine VT_1_ and VT_2_. Participants were categorised as responders to the intervention if an absolute VT_2_ change of at least 1.9% was attained. Thirty-eight participants were included in the analysis. A significant difference in VT_2_ change was found between individualised (pre vs. post: 70.6% vs. 78.7% maximum oxygen uptake (VO_2_max)) and standardised (pre vs. post: 72.5% vs. 72.3% VO_2_max) exercise groups. Individualised exercise prescription was significantly more efficacious (*p* = 0.04) in eliciting a positive response in VT_2_ (15/19, 79%) when compared to the standardised exercise group (9/19, 47%). Individualised exercise prescription appears to be more efficacious than standardised exercise prescription in eliciting a positive VT_2_ change among physically inactive adults. Increasing VT_2_ allows for greater tolerance to higher exercise intensities and therefore greater cardiovascular health outcomes.

## 1. Introduction

Extensive research has presented the enhanced health benefits of performing higher intensities of exercise [1]. High-intensity exercise participation has been demonstrated to promote greater cardiovascular health benefits when compared to low intensity exercise [2,3]. Indeed, exercise intensity has been established as the single most important aspect of aerobic exercise prescription in reducing cardiovascular death risk and all-cause mortality [4]. Weekly participation in a single bout of high-intensity exercise has been recognised to significantly reduce cardiovascular disease risk [4]. No additional benefit was found in increasing the duration or number of sessions per week [4]. Furthermore, high-intensity exercise is recognised to be more effective, when compared to lower exercise intensities, at reducing metabolic syndrome (MetS) disease risk, a condition associated with a 60% increase in cardiovascular disease (CVD) risk [5]. Previous literature established the risk of developing MetS could be decreased by 3–10% through regular participation in moderate to vigorous physical activity [6]. No significant reduction in MetS risk was recognised among those who participated in light physical activity [6]. Ultimately, such profound impact high-intensity exercise has on reducing overall CVD risk is thought to be a result of its superiority in improving cardiorespiratory fitness (CRF), as measured by peak or maximum VO_2_ (VO_2_peak or VO_2_max) [7,8,9]. Well-documented research highlights high-intensity exercise as being the most effective training method in improving CRF when compared to alternate exercise intensities [7,8,9]. Increased exercise participation at vigorous to high intensities has also been associated with greater improvements in glucose utilization, as well as significant improvements in insulin stimulated suppression of adipose tissue lipolysis [10]. Ultimately, a high second ventilatory threshold (VT_2_) allows for a greater capacity to perform higher exercise intensities, and therefore better health outcomes.

The VT_2_ is recognised as the point during incremental exercise at which an exponential increase in minute ventilation (V_E_) relative to VO_2_ (oxygen uptake) occurs [11]. The steep climb in V_E_, a product of respiratory rate and tidal volume, is driven by the heightened carbon dioxide (CO_2_) production as a consequence of hydrogen ion accumulation as exercise intensity progresses [12]. VT_2_ signifies the point between easily sustained exercise and exercise that is only able to be sustained for a short period of time. Ultimately, VT_2_ has been established as an important indicator of overall exercise tolerance [1,12,13,14]. Specifically, it is recognised as a good marker of one’s ability to perform higher intensities of exercise for longer durations [13].

It is well recognized that exercising at a high intensity leads to greater improvements in VT_2_ [15]. Commonly, two forms of exercise intensity prescription are used among athletic and clinical populations, namely, standardised and individualised [16]. Standardised exercise prescription presents as the most common form and is recognised as prescribing exercise based on a relative percentage of a physiological parameter such as heart rate (i.e., percentage of heart rate reserve [HRR] or heart rate maximum) [16]. Comparatively, individualised exercise prescription is derived from individual physiological thresholds such as VT_2_ and VT_1_ (the first ventilatory threshold) [17]. VT1 is defined as the initial increase in VE during incremental exercise, indicating the boundary between low- to moderate-intensity exercise [12]. Indeed, individualised exercise prescription has been suggested to be more effective in eliciting an overall positive training response in comparison to standardised exercise prescription. Specifically, Wolpern et al. [17] presented that 100% of apparently healthy sedentary participants prescribed with individualised exercise demonstrated a statistically significant increase in overall relative VO_2_max in contrast to only 41.7% of those prescribed with standardised exercise prescription. However, to the authors knowledge, research thus far has yet to directly investigate the effects of change in VT_2_ measures following individualised and standardised exercise prescription among physically inactive adults.

The aim of this study was to compare two approaches to aerobic exercise intensity prescription (standardised versus individualised) on VT_2_ among physically inactive adults over a 12-week exercise intervention. It was hypothesised that individualised exercise prescription would be more effective in increasing VT_2_ compared to standardised exercise prescription in physically inactive adults.

## 2. Materials and Methods

### 2.1. Research Design and Methodology

The study recruited physically inactive males and females interested in improving their overall health and wellness. The present study is a sub-study of a previously published investigation [18], reporting on VT_2_ as a primary outcome. Only those who had at least 70% adherence to the exercise interventions were included in the analysis of this sub-study. Participants were recruited from a community wellness program via advertisement at the local university and newspaper. To meet the inclusion criteria for the investigation, participants were required to be currently completing less than 30 min of physical activity on three days a week or less and be aged between 30–75 years. Participants completed the International Physical Activity Questionnaire (IPAQ) to identify physical activity levels and confirm study eligibility [19]. Participants were allowed to clarify any questions to ensure correct inclusion criteria was met. In order to ensure participant safety, individuals were required to undergo a standard medical history questionnaire and an intake interview. Participants who identified signs or symptoms consistent with pulmonary, cardiovascular or metabolic conditions within the screening were excluded from the investigation. Participants were also required to verbally confirm that they would not alter their regular nutritional intake habits throughout the intervention. To monitor nutritional intake, participants were instructed to complete a nutritional logbook, detailing their nutritional intake in the previous three days. This occurred at pre-and post-exercise intervention testing. Nutritional information sourced within the 3-day dietary logs (two weekdays and one weekend day) included the type and quantity of food consumed, brand of food, how it was prepared and the time of day of consumption. This allowed the most accurate energy intake, percentage of macronutrients and grams of micronutrients consumed to be analysed. Written informed consent was obtained from all study participants prior to initiation of the study. A full description of the randomized control trial methodology and rationale has been previously published in detail [20]. The study ethics were approved by the Auckland University of Technology Ethics Committee (16/264) and Western State Colorado University Institutional Review Board (HRC2016-01-90R6).

Experimental testing was conducted at baseline and post 12-week exercise intervention. In order to decrease data variability, pre- and post-exercise experimental testing was completed on the same time and day of the week. Participants were instructed to avoid consumption of food and strenuous exertion for 12 h prior to testing. The post experimental assessment took place 2–3 days following the last exercise training session.

### 2.2. Ventilatory Threshold

Upon arrival at the laboratory and 20–30 min prior to completing a graded exercise test (GXT), basic anthropometric and resting HR values were assessed. Resting HR was assessed following five minutes of seated rest with back support, feet on the ground, and arms supported near heart level in a distraction free assessment room. After the 5 min of rest, a medical-grade pulse oximeter (Nonin Medical Inc., Plymouth, MN, USA) was used to establish a resting HR measure. Subsequently, participants were weighed to the nearest 0.1 kg on a medical grade scale and height was measured to the nearest 0.5 cm using a stadiometer (Tanita Corporation WB-3000, Tokyo, Japan).

To determine VT_2_, a graded exercise test (GXT) to exhaustion was conducted through implementation of a modified Balke pseudo-ramp protocol on a motorized treadmill (Powerjog GX200, Portland, ME, USA). A 4 min warm-up was conducted for each participant at a self-selected speed and incline of 0%. Following warm-up, the treadmill incline was increased by 1% every minute until exhaustion. To monitor heart rate (HR), a chest strap and radio telemetric device (Polar Electro, Woodbury, NT, USA) was used. Expired oxygen (O_2_) and CO_2_ exchange data were analysed using a metabolic analyser (Parvo Medics TrueOne 2.0, Salt Lake City, UT, USA). The metabolic analyser was calibrated against a known gas mixture (16% O_2_ and 4% CO_2_) and room air (20.93% O_2_ and 0.03% CO_2_), as outlined in the manufacturer’s instruction manual. The final two highest 15 s epochs were averaged to represent the participants’ VO_2_max. To ascertain that the participant completed a maximal exercise test, a verification protocol was implemented. This verification procedure has been established as a robust and accurate measure to indicate if participants have indeed achieved a VO_2_max value [21]. Such protocol involved instructing the participants to complete a four-minute warm-up, 20 min after the completion of the GXT, followed by a volitional test to exhaustion at a constant workload that was 5% higher than the last completed stage of the GXT. The constant workload used for the verification procedure was determined by increasing the speed, incline, or combination of these to achieve a 5% higher MET value than the maximal MET value achieved during the GXT. Participants were encouraged to complete a duration greater than two minutes. The VO_2_max achieved during the verification protocol was also calculated by taking the average of the highest 15 s epochs. A maximal exercise test was confirmed if the two maximal VO_2_ values were within 3% of each other, since this is recognised as the measurement error of the metabolic analyser. If the discrepancy was greater than 3%, the participants were asked to repeat the GXT and verification protocol 24–72 h following until confirmation of a maximal exercise test was achieved [22]. The highest measured HR during the GXT was recognised to be the participants maximal HR. Heart rate reserve (HRR) was then calculated by subtracting the difference of HRmax obtained during the maximal GXT and resting HR assessed 20–30 min prior to GXT, as described previously.

The first ventilatory threshold (VT_1_) was necessary to identify to guide exercise prescription among the individualised group. This measure denotes the point between light to moderate exercise intensity and was identified as the point at which VE/VO_2_ increased with no subsequent increase in VE/VCO_2_ as determined by the metabolic analyser (Parvo Medics TrueOne 2.0, Salt Lake City, UT, USA). VT_2_ was identified as the point where VE/VO_2_ and VE/VCO_2_ simultaneously increased, determined by the metabolic analyser software (Parvo Medics TrueOne 2.0, Salt Lake City, UT, USA). This VT analysis technique has been used in numerous similar studies [15,23,24]. VT_2_ was expressed as a percentage of absolute VO_2_max.

### 2.3. Exercise Prescription

**Figure 1:**
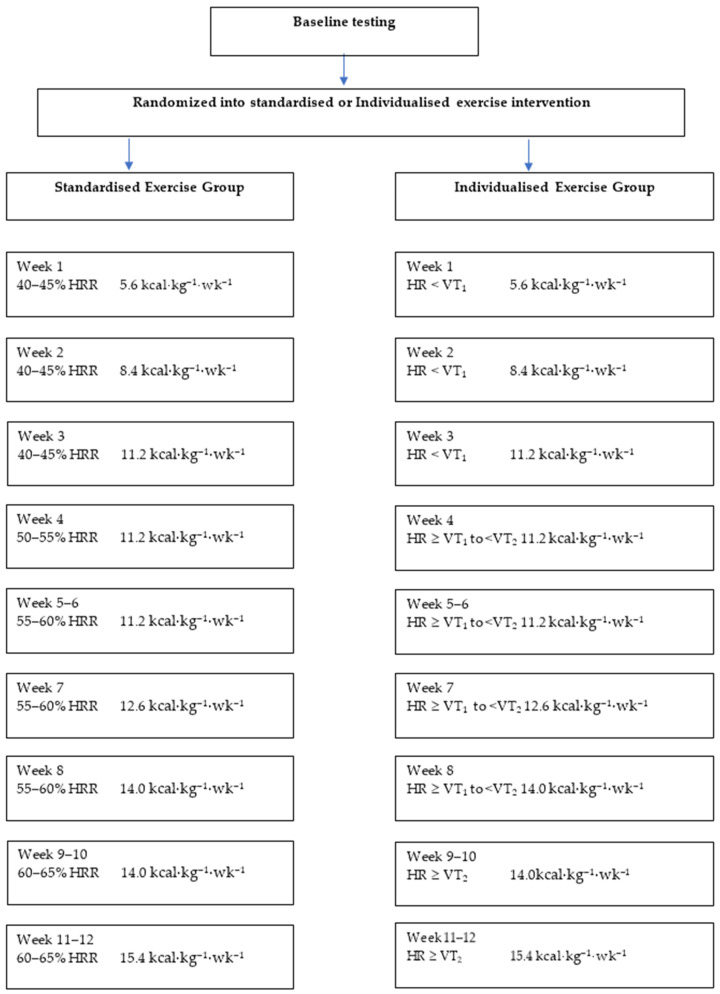
Exercise prescription progression among both iso-caloric exercise intervention groups throughout the intervention. HRR: Heart Rate Reserve; HR: Heart rate; VT_1_: Ventilatory threshold 1; VT_2_: Ventilatory threshold 2; Kcal: kilocalories.

Participants were randomised into either standardised exercise prescription or individualised exercise prescription (Figure 1). This was achieved by using a computer-generated sequence of random numbers stratified by sex. Following randomisation into one of the two exercise interventions participants exercised three times a week for 12 weeks.

Exercise workload for the standardised exercise group was prescribed according to the American College of Sports Medicine (ACSM) guideline [16], as a target heart rate (HR) range calculated as percentage of HRR using the following equation:

(1) Target HR range=[(MaximalHR−RestingHR)∗Desired%HR]+RestingHR

Exercise intensity within the individualised exercise group was calculated based off HR ranges relative to VT_1_ and VT_2_ calculated during the GXT. Participants within the individualised exercise group had their exercise intensity zones established through the following equations according to the American Council on Exercise (ACE) guidelines [17]:

- Target HR  < VT_1_  =  HR range: 10 beats per minute (bpm) below VT_1_ to the HR at VT_1_
- Target HR  ≥  VT_1_ to  <  VT_2_  =  HR range: 15 bpm above VT_1_ to below VT_2_
- Target HR  ≥  VT_2_ = HR range: Heart rate at VT_2_ to 10 bpm above VT_2_

Figure 1 outlines the progression in overall exercise intensity of the exercise intervention groups. Exercise volume of both groups was calculated based on energy expenditure per kg of body weight per week (kcal·kg^−^^1^·wk^−^^1^) to ensure exercise volume was matched between both interventions. The energy expenditure was determined by associating the exercise HR prescribed with the energy expenditure derived from the maximal GXT. Body weight (kg) was measured via medical grade scales to the nearest 0.1 kg.

### 2.4. Exercise Training

Upon arrival for the prescribed exercise sessions, participants were instructed to rest in a seated position for five minutes. After five minutes of seated rest, resting HR and blood pressure were measured and recorded to ensure it was safe for the participants to perform exercise and used to monitor recovery following cessation of the exercise session. Blood pressure was measured on the participants left arm using a stethoscope and sphygmomanometer (American Diagnostic Corporation Diagnostic 700 Series, Hauppauge, NY, USA). Depending on preference and availability, participants completed exercise sessions on either a motorised treadmill, elliptical training and/or a stationary bike. In order to limit fluctuations in acute exercise intensities compared to prescribed intensity, participants were limited to 5 and 10 min for use of the stationary bike and elliptical trainer, respectively, with the majority of the exercise occurring with the use of a motorized treadmill. This variety in modality was implemented to increase enjoyment and mimic a community wellness program setting. Participants were instructed to warm-up at a self-selected pace and workload for five minutes, at which point the prescribed exercise intensity was reached and the participant felt ready to begin. The participants worked at such intensity for the calculated exercise duration, based on values obtained during the GXT with continuous monitoring (1/3 and 2/3 of the total time of exercise) of HR through use of a chest strap and radiotelemetry receiver (Polar Electro, Woodbury, NY, USA). This method helped ensure the correct prescribed exercise intensity was being used throughout the session. Following the workload period, participants performed a five-minute cool-down with decreasing workload until HR returned to within 15 bpm of resting HR.

### 2.5. Data Management and Analysis

Data analysis was completed using SPSS version 25.0. Assumption of normality was confirmed through use of the Shapiro–Wilk tests. Analysis of VT_2_ change was based on individual responses and compared to a determined threshold of a clinically meaningful change. The threshold of individual change of VT_2_ was assessed based on a similar methodology investigating six concepts of lactate threshold (a similar measure of VT_2_) to calculate the subject coefficient of variation, recognised as 1.9% [25]. Such value could categorise each participant as either a likely responder or likely non-responder. Participants who demonstrated a VT_2_ change of ≥1.9%, deemed to indicate a meaningful change, were categorised as likely responders, while those who demonstrated a change of <1.9% were categorized as likely non-responders with no clinically meaningful change in VT_2_. A repeated measures ANCOVA was used to analyse the between-group differences of change in VT_2_ and other continuous physiological variables collected from baseline to post intervention. Baseline values were assigned as the covariate, while the dependent variable was recognised as the post-exercise intervention values. The Chi-square test was used to analyse the proportion of responders to a clinically meaningful change in VT_2_ between groups. Statistical significance was set at *p* < 0.05.

## 3. Results

**Figure 2:**
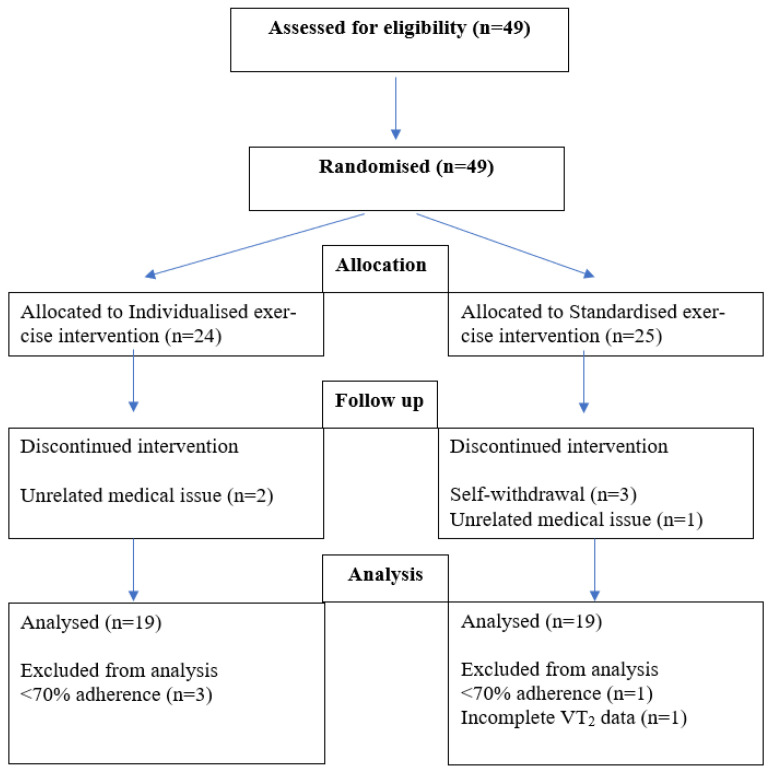
Number of participants recruited and rationale for data analysis exclusion.

Of the 49 participants recruited, a total of 11 withdrew or were not included in the analysis. This was due to unrelated medical issues (*n* = 3), falling below 70% adherence (*n* = 4), self-withdrawal (*n* = 3), and incomplete pre- and post-intervention VT_2_ data (*n* = 1) (Figure 2). Consequently, 38 participants were included within the final data analyses. The standardised exercise intervention group recorded a mean adherence across the 12 weeks of 82.9% ± 5.7%, while the individualised exercise intervention group recorded a mean adherence of 86.1% ± 4.7%.

Mean pre-and post-exercise intervention parameter characteristics are presented in Table 1. A significant between group difference in VO_2_max was noted at baseline. The individualised exercise group recorded a mean absolute VO_2_max of 2.0 ± 0.6 (L·$\min^{-1}$), while the standardised exercise group recorded a mean of 2.4 ± 0.8 (L·$\min^{-1}$). There was no significant change in mean caloric intake from pre- to post-exercise intervention between groups (*p* > 0.05).

### 3.1. Changes in VT_2_

VT_2_ was expressed as a percentage of absolute VO_2_max (L/min), as presented by previous studies [15,26,27]. A repeated measures ANCOVA analysis indicated a statistically significant difference in VT_2_ change at post exercise intervention (*p* < 0.001) between training groups. Following the 12-week exercise intervention, VT_2_ within the individualised group demonstrated a mean improvement from pre- to post-exercise intervention of 70.6 ± 8.5% to 78.7 ± 6.0%, which was significantly greater in comparison to the standardised exercise group 72.5 ± 7.8% to 72.3 ± 5.5%.

### 3.2. Incidence of VT_2_ Responders and Non-Responders

**Figure 3:**
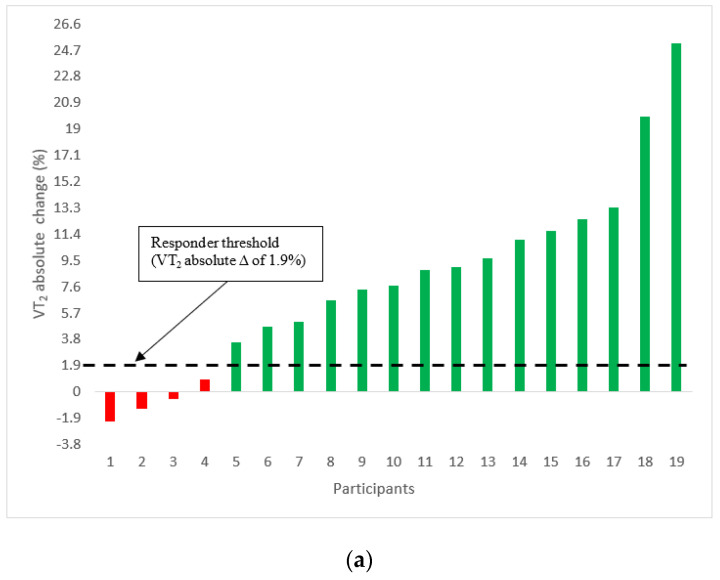

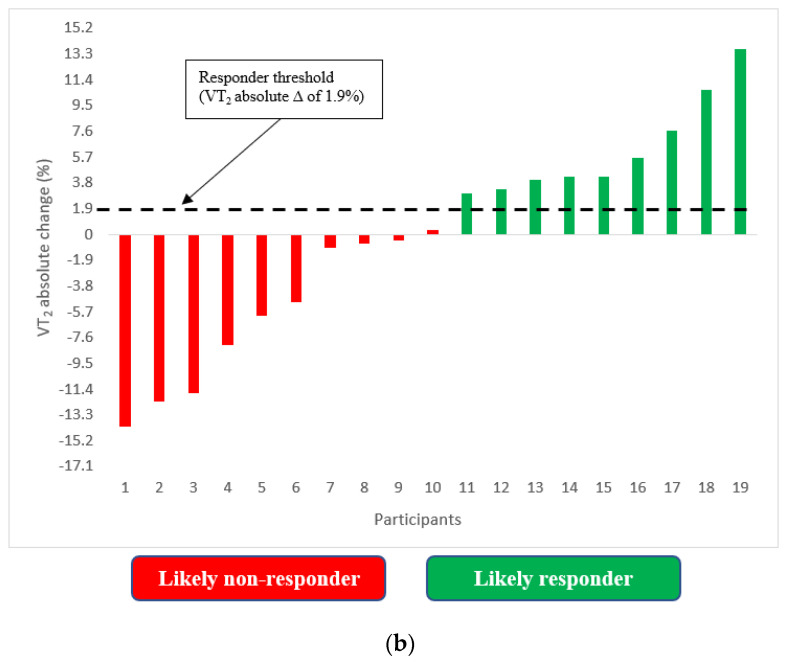
VT_2_ absolute change post individualised (**a**) and standardised (**b**) exercise intervention.

The proportion of likely responders and non-responders to an absolute change in VT_2_ expressed as a percentage of VO_2_max is presented in Figure 3a,b. There were 15/19 (79%) likely responders to a significant meaningful change in VT_2_ in the individualised exercise group, while only 9/19 (47%) participants in the standardised exercise group were recognised as likely responders. A Chi-square test indicated a significant difference in the proportion of responders between the two training groups (*p* = 0.04).

## 4. Discussion

The main aim of this study was to compare the efficacy of individualised and standardised exercise prescription on overall VT_2_ change among physically inactive adults. As hypothesised, the individualised exercise prescription was more effective in increasing VT_2_ compared to standardised exercise prescription in physically inactive adults. This is supported by our sub-analysis, which showed that the individualised exercise group resulted in significantly more likely responders (15/19, 79%) to a positive change in VT_2_ (>1.9%) from pre- to post-intervention, when compared to the standardised exercise intervention group (10/19, 47%). To the best of the authors’ knowledge, this is the first study to implement a specific threshold for VT_2_ response and directly compare standardised and individualised exercise prescription on direct VT_2_ change among physically inactive adults. The findings of this study are of significance as increases in VT_2_ could lead to an improved ability to tolerate higher intensities of exercise [26]. Such outcome is directly recognised to improve one’s functional ability to perform activities of daily living, cardiovascular health, and overall all-cause mortality [7,8,28].

Previous literature has established that participants who take part in higher intensities of exercise demonstrate a greater change to VT_2_ in comparison to those who participate in lower intensity exercise training [15,27]. More specifically, exercise conducted at an intensity near or at VT_2_ is an adequate training stimulus to improve VT_2_ among physically inactive individuals [29]. This could possibly explain why the individualised approach to exercise prescription has shown to be more efficacious in eliciting a favourable VT_2_ response relative to a standardised exercise prescription in the present study.

Previous literature has also supported the use of individualised exercise prescription in eliciting a positive training response among various cardiovascular health measures [18,30]. Specifically, individualised exercise prescription is recognised to have a pronounced positive impact on CRF when compared to standardised exercise prescription [30]. Key markers of cardiovascular health and disease risk have also been established to be positively influenced by individualised exercise prescription, significantly more so than standardised exercise prescription [18].

It has been suggested that a standardised approach, using relative percent methods to exercise prescription fails to consider individual variability in the training stimulus that is necessary to elicit a positive VT_2_ response. For instance, it has been found that the required training stimulus to improve VT_2_ is usually achieved at 78–93% of an individual’s maximal HR and/or 70–93% of HRR among apparently healthy individuals [31]. Current ACSM (American College of Sports Medicine) exercise guidelines recommend adults aged 18–65 years to complete 150 min of physical activity a week at an intensity of 57–76% HRmax [16]. Such a broad range of standardised exercise guidelines suggests that the minimum limit of recommended intensity does not provide an adequate training stimulus necessary to elicit a positive VT_2_ change [31]. The individual variance in training response has been proposed to be attributed to numerous cardiovascular components including muscle oxidative capacity, cardiac output and arteriovenous O_2_ difference, components which are notably influenced by age and lifestyle factors [32]. Genetics has also been recognised to have a significant influence in training response among individuals [33]. Specifically, Sarzynski, Gosh and Bouchard [34] accounts 47% of total trainability variance to genetics. However, the specific molecular mechanisms behind such variance remain poorly understood, as well as the remaining factors which may determine the variance in training response [34].

A range of mechanisms have been presented as to why exercise at a higher intensity leads to a greater training response among individuals. High-intensity exercise is recognised to have a significant impact on the intracellular signalling sequence [35]. This is largely subject to the increased overall energy requirements of high-intensity exercise in comparison to light and moderate exercise intensity [36]. Such an increase in energy demand creates greater enzymatic activity involved in energy production during exercise [36]. This stimulus has been established to directly regulate mitochondrial biogenesis and subsequently lead to improvements in CRF and exercise tolerance [35,36,37]. In fact, Di Donato et al. [38] presented that the greater the exercise intensity, the greater the rate of mitochondrial biogenesis. Further research additionally recognises that such increase in enzyme activity leads to improved buffering of hydrogen ions within the skeletal muscle [39]. This adaptation prolongs anaerobic metabolism and therefore leads to greater tolerance of exercise at heightened exercise intensities, as well as greater tolerance of submaximal exercise [26,40]. Additionally, the shear stress induced by high-intensity exercise participation triggers a greater cellular and molecular response related to endothelial function [35]. Marsh and Coombes [41] attributes this improved endothelial function to the increase in endothelial production of nitric oxide (NO). Cellular stress produced by high-intensity exercise promotes the release of extracellular superoxide-dismutase, which encourages the production of NO [42]. NO is recognised to induce vascular vasodilation, inhibit platelet aggregation and consequently reduce progression and long-term risk of vascular disease [41,43]. Such adaptations also correlate to improved O_2_ delivery capacity and therefore greater exercise tolerance [35]. Ultimately, these mechanisms may help explain the greater increase in VT_2_ that occurs following higher intensity exercise when compared to lower exercise intensities [35,36,37,39]. Therefore, prescribing exercise at greater intensities specific to individual thresholds (individualised exercise prescription), greater cellular and metabolic stress is created and consequently greater VT_2_ adaptations.

## 5. Strengths and Limitations

There are a range of limitations within the study which must be acknowledged. Participants were able to choose three different modalities for exercise training session. While time limitations were placed on the stationary bike and elliptical trainer, it is recognized these modes may induce differing metabolic considerations compared to exercise completed on the motorised treadmill. The absence of a control group within the study removes the ability to confirm that the VT_2_ changes between the training groups are in fact directly influenced by the specific training intervention. To minimise such limitation, all variables were controlled to the best of the testers’ abilities. Specifically, participants were instructed not to alter their dietary intake throughout the exercise intervention. Participants completed a nutritional logbook detailing their nutritional intake in the previous three days at pre and post intervention. The use of caloric energy expenditure calculations ensured exercise volume between the two exercise intervention groups was kept equal. Pre- and post-exercise experimental testing was also completed at the same time and day of the week and within 2–3 days of completing the 12-week exercise intervention. Additionally, a limited representation of men (24%) within the study may have impacted the overall accuracy of the results in representing the general population. The mean age difference between participants within the individualised and standardised exercise intervention group (+7.37 years) must also be acknowledged to have the potential to skew results. Age has been associated as a factor that influences VT_2_ [32]. Such variance in age between the two groups may therefore impact incidence of response among groups. This study did not implement a crossover study design, which could have provided information regarding the response of participants within different exercise interventions. However, the implementation of a crossover design would require a washout period, which could have removed positive health-promoting behaviour, so this was not introduced due to ethical considerations. This study is one of the first to implement a VO_2_max verification protocol. The use of such protocol confirmed participants completed a maximal GXT, ensuring the most accurate VT_2_ values are reported. Finally, the use of a VT_2_ coefficient of variation allowed overall meaningful incidence of response to be measured, further improving the overall accuracy of the reported results [25].

## 6. Conclusions

The results of this study demonstrate that individualised exercise prescription appears to be more efficacious in eliciting positive improvements in VT_2_ in comparison to standardised exercise prescription when exercise is anchored by HRR measures. Findings from this study support and add to the knowledge surrounding the promotion of individualised exercise prescription in overall training responsiveness. Such study is however, the first of its kind to specifically compare individualised and standardised exercise prescription on VT_2_ change. While individualised exercise intensity prescription has shown to improve VT_2_ more so than standardised exercise intensity prescription, more research into the optimal dosage of exercise (duration and frequency) would prove greatly beneficial. Such research will help further guide the clinical practice of health professionals to elicit greater positive VT_2_ change and thus greater health outcomes in individuals.

## Figures and Tables

**Table 1:** Pre- and post-mean outcome measures for standardised and individualised exercise groups.

Parameters	Standardised (*n* = 19; Women, *n* = 15; Men, *n* = 4)		Individualised (*n* = 19; Women, *n* = 14; Men, *n* = 5)		Between-Group Difference
	Pre	Post	Pre	Post	*p*-Value
**Age**	52.3 ± 11.7	-	45.0 ± 11.3	-	-
**Height**	168.3 ± 9.6	-	172.2 ± 7.0	-	-
**Weight**	84.7 ± 21.1	84.2 ± 20.2	80.2 ± 15.5	80.3 ± 15.6	-
**VT2 (% VO_2_max)**	72.5 ± 7.8	72.3 ± 5.5	70.6 ± 8.5	78.7 ± 6.0 *	<0.001
**Absolute VO_2_ at VT_2_ (L/min)**	1.5 ± 0.5	1.6 ± 0.5	1.7 ± 0.5	2.0 ± 0.7 *	0.017
**Relative VO_2_ at VT2 (mL/kg/min)**	17.3 ± 3.5	18.7 ± 3.1	20.8 ± 5.3	24.7 ± 7.1 *	0.016
**VO_2_max (L/min)**	2.0 ± 0.6	2.2 ± 0.6	2.38 ± 0.8	2.5 ± 0.8	0.579
**VO_2_max** **(mL/kg/min)**	24.0 ± 4.7	25.9 ± 4.8	29.7 ± 7.8	31.4 ± 8.5	0.635
**Daily caloric intake (kcal)**	1519 ± 563	1518 ± 500	1539 ± 493	1555 ± 403	0.967

* Statistically significant (*p* < 0.05) difference at post-intervention between groups.

## Data Availability

The data presented in this study are available on request from the corresponding author.
